# The profile of abstract rule learning in infancy: Meta‐analytic and experimental evidence

**DOI:** 10.1111/desc.12704

**Published:** 2018-07-16

**Authors:** Hugh Rabagliati, Brock Ferguson, Casey Lew‐Williams

**Affiliations:** ^1^ School of Philosophy, Psychology and Language Sciences University of Edinburgh Edinburgh UK; ^2^ Department of Psychology Northwestern University Evanston Illinois; ^3^ Department of Psychology Princeton University Princeton New Jersey

## Abstract

Everyone agrees that infants possess general mechanisms for learning about the world, but the existence and operation of more specialized mechanisms is controversial. One mechanism—rule learning—has been proposed as potentially specific to speech, based on findings that 7‐month‐olds can learn abstract repetition rules from spoken syllables (e.g. ABB patterns: *wo‐fe‐fe*,* ga‐tu‐tu*…) but not from closely matched stimuli, such as tones. Subsequent work has shown that learning of abstract patterns is not simply specific to speech. However, we still lack a parsimonious explanation to tie together the diverse, messy, and occasionally contradictory findings in that literature. We took two routes to creating a new profile of rule learning: meta‐analysis of 20 prior reports on infants’ learning of abstract repetition rules (including 1,318 infants in 63 experiments total), and an experiment on learning of such rules from a natural, non‐speech communicative signal. These complementary approaches revealed that infants were most likely to learn abstract patterns from meaningful stimuli. We argue that the ability to detect and generalize simple patterns supports learning across domains in infancy but chiefly when the signal is meaningfully relevant to infants’ experience with sounds, objects, language, and people.

**RESEARCH HIGHLIGHTS**

- Abstract rule learning—the ability to find generalizable patterns in incoming perceptual input—has been proposed as a mechanism that supports learning in infancy.
- We conducted a meta‐analysis of 20 papers on infants’ learning of abstract repetition rules (including 1,318 infants in 63 experiments), plus an experiment that substantiated its key finding.
- Infants were most likely to detect and generalize patterns from stimuli that are meaningfully relevant to their everyday experience with sounds, objects, language, and people.
- Our complementary meta‐analytic and experimental approaches provide the first coherent explanation for the diverse, messy, and occasionally contradictory findings from research on infant rule learning.

## 1 INTRODUCTION

Just as scientists draw inferences by combing through disorderly data, so infants learn by sifting the most important information out of a noisy signal. How they achieve this is currently unclear. While all modern theories of cognitive development agree that infants possess powerful general mechanisms for learning about the world (Csibra & Gergely, 2009; Doumas, Hummel, & Sandhofer, 2008; Endress & Bonatti, 2016; Frost, Armstrong, Siegelman, & Christiansen, 2015; Gopnik, 2012; Marcus, 2000; Romberg & Saffran, 2010; Saffran & Kirkham, 2017; Smith, Suanda, & Yu, 2014), the ways in which these mechanisms are narrowed and specialized, to focus on extracting the most vital information from different specific domains, is much more controversial.

One possibility is that, alongside their domain general capacities, infants possess a set of learning mechanisms that are specialized for extracting information from and about very specific domains, such as language. Evidence for this comes from studies on how infants extract and generalize across regularities (Gerken, 2006; Marcus, 2000; Marcus, Vijayan, Bandi Rao & Vishton, 1999), including a prominent finding that such learning seems to be facilitated by speech. In particular, Marcus and colleagues have shown that 7‐month‐old infants have no difficulty learning what they call an abstract rule, like the repeating patterns ABB, AAB or ABA, when it is instantiated in sets of syllables (e.g. *wo‐fe‐fe* and *ga‐tu‐tu* follow an ABB pattern).^1^ However, infants of the same age fail to learn the same abstract patterns from closely matched stimuli, such as sequences of animal sounds, tones, or musical notes (Marcus et al., 1999; Marcus, Fernandes & Johnson, 2007). The topic of how infants learn these abstract patterns has since become a mainstay for developmental and cognitive science.

Some of this research has cast some doubt on Marcus et al.'s interpretation; many findings now provide evidence that infants can learn some abstract repetition rules from some non‐speech stimuli under some conditions. For instance, Thiessen (2012) and Frank, Slemmer, Marcus, and Johnson (2009) found that infants were able to learn such rules from multimodal stimuli. Dawson and Gerken (2009) showed that infants could learn rules from tones when they were 5 months old, but failed to do so at 7 months. Ferguson and Lew‐Williams (2016) revealed that 7‐month‐olds learn rules from tones, but only if the infants have been given a prime in which the tones are treated as a meaningful communicative signal, while Saffran, Pollak, Seibel, and Shkolnik (2007) showed that infants learn rules from sequences of meaningful and categorizable pictures, like animals.

While this collection of results does provide evidence that infants can learn abstract repetition rules from stimuli other than speech, the literature has yet to provide a satisfying theoretical explanation for the set of stimuli and conditions under which learning can occur. One possibility is that there is no easily circumscribable set of *necessary* conditions under which infants can learn these rules, but rather a variety of *sufficient* conditions. For example, it could be that infants find it easier to learn repetition rules from a diverse range of stimuli when they are younger (Dawson & Gerken, 2009), or infants might learn from any stimuli that are particularly meaningful to them, where that is defined by a variety of factors including communicative intent, ecological relevance, and classifiability (Ferguson & Lew‐Williams, 2016; Saffran et al., 2007). But another possibility is more concerning: that the published positive results may, in part, reflect noise and bias in the scientific literature.

There are a number of reasons to be concerned about the signal‐to‐noise ratio in published work on infant cognition (Frank et al., 2017), especially regarding the phenomenon of abstract rule learning. Most importantly, infant studies are usually underpowered: they use small sample sizes and noisy dependent measures. In addition, independent replications are rare, laboratories differ in their protocols for conducting studies, and researchers frequently have to make subjective decisions about data (e.g. whether to exclude a fussy participant). Finally, publication bias is likely to be particularly problematic for this literature: Since Marcus et al.'s (2007) demonstration of domain specificity (i.e. a large and high‐powered null result when learning from non‐speech sounds), the incentives for publication will have favoured reports that contain positive findings. Each of these conditions could potentially result in an elevated level of false positives. Consistent with these worries, there are indeed discrepancies between published findings in the literature. For example, while Dawson and Gerken (2009) found that 5‐month‐olds could learn abstract repetition rules from tones, Frank et al. (2009) found that 5‐month‐olds could not learn repetition rules from speech. While Johnson et al. (2009) and Thiessen (2012) found evidence for learning via preferences to view or listen to a familiar pattern, most other studies found evidence for learning via novelty preferences. And a number of studies (e.g. Johnson et al., 2009; Rabagliati, Senghas, Johnson, & Marcus, 2012) have reported that infants are able to learn some patterns but not others, for example, succeeding on ABB but failing on AAB. While each of these inconsistencies alone can be explained away (perhaps the null result in Frank et al., 2009, was a false negative, perhaps some patterns are genuinely easier to learn than others), in combination they raise the possibility that at least some of the claims found in this literature may be incorrect.

This paper takes two routes to evaluating the reliability of abstract rule learning: a meta‐analysis that aggregates the experimental evidence on this topic, and an experiment that replicates and extends key findings of that meta‐analysis. Our meta‐analysis not only allowed us to test the statistical reliability of infant learning, following concerns about replicability, but also assessed the most prominent proposals as to what factors moderate learning of abstract repetition rules. We tested whether learning is facilitated just for spoken syllables (Marcus et al., 2007), or for any stimulus that is particularly meaningful to the infant, building on suggestions that infants learn better from stimuli that are communicatively relevant (Ferguson & Lew‐Williams, 2016) or that are ecologically relevant (e.g. are drawn from familiar categories, as in studies by Saffran et al., 2007). Finally, we also tested whether younger infants are able to acquire abstract repetition rules from a more diverse set of stimuli than older infants (Dawson & Gerken, 2009), and whether some patterns are easier to learn than others (Johnson et al., 2009; Rabagliati et al., 2012).

### 1.1 Meta‐analysis of infant rule learning

We aggregated and evaluated the evidence from 20 papers on how infants learn abstract repetition rules, hewing closely to procedures that have been successfully used to investigate other infant language phenomena (Bergmann & Cristia, 2016; Bergmann et al., 2018; Lewis et al., submitted; Tsuji & Cristia, 2014), including studies on artificial grammar learning (Black & Bergmann, 2017; Cristia, 2018). We followed the ‘Preferred Reporting Items for Systematic Reviews and Meta‐Analyses’ (PRISMA) statement (Moher, Liberati, Tetzlaff, Altmann, & PRISMA Group, 2009) in preparing this meta‐analysis. A PRISMA checklist can be found at https://osf.io/5k3vw/, which also contains links to the databases we created. We augmented our meta‐analysis with *p*‐curve analyses that test for the presence of publication bias in a literature.

## 2 METHODS

### 2.1 Report identification

We identified a pool of published journal articles, conference proceedings, theses, and other unpublished reports. These were drawn from papers already known to the authors (20 reports), personal communications with expert researchers (27 reports, including some duplicates), calls for data via the info‐childes and CogDevSoc listservs (two additional reports not uncovered through other searches), and three Google Scholar searches (1,522 reports, including many duplicates). Google Scholar searches were conducted on 2 July 2017. One was a basic search using the terms *infant*, ‘*rule learning*’, and ‘*abstract rule learning*’; one searched for articles citing Marcus et al. (1999) and used the terms *infant* and *mean* (intended to distinguish empirical reports from reviews); and one searched for articles citing Marcus et al. (1999) and used only the term *infant*.

### 2.2 Report selection

**Figure 1:**
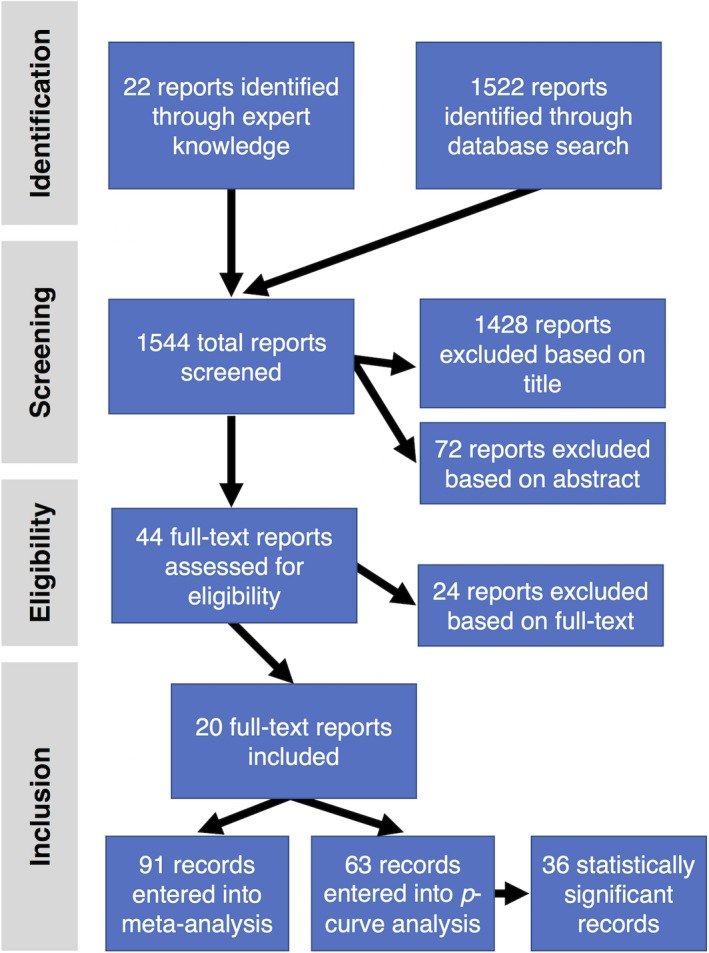
A PRISMA‐style flow chart for report identification and exclusion procedure. See main text for full details

We included reports that had the following characteristics: (i) Participants were typically developing infants under 24 months of age, (ii) participants were exposed to strings of three stimuli generated from a single repetition pattern (e.g. AAB, ABB), and (iii) participants were tested on their behavioural response to new strings generated from either the familiarized pattern (Familiar pattern trials) or a novel pattern (Novel pattern trials). We thus excluded reports that used different paradigms or asked somewhat different questions, including neuroimaging studies (e.g. Gervain, Macagno, Cogoi, Peña, & Mehler, 2008), relational‐match‐to‐sample studies (e.g. Tyrell, Stauffer, & Snowman, 1991), studies that did not use repetition patterns (e.g. Koulaguina & Shi, 2013), and studies in which infants were exposed to two patterns (e.g. Hochmann, Benavides‐Varela, Nespor, & Mehler, 2011; Kovács & Mehler, 2009). While these studies would merit inclusion when reviewing the rule learning literature, their differences would introduce undue noise to our statistical analyses. Figure 1 shows a PRISMA‐style flowchart for our identification and exclusion process.

This left us with 20 reports in total (Table 1), comprising 17 published journal articles, one conference proceeding paper, and two Master of Science theses. The reports were drawn from 11 different labs.

**Table 1:** Reports included in the present meta‐analysis. Number of records refers to number of records in the meta‐analysis database

Authors	Year	Ages	Stimuli	Number of participants	Number of records	Peer Reviewed
Bahmann & Levelt	2016	7	Speech	10	1	no
Bulf, Brenna, Valenza, Johnson, & Turati	2015	7	Faces	71	2	yes
Bulf, de Hevia, Gariboldi, & Macchi Cassia	2017	7	Abstract shapes	64	1	yes
Dawson & Gerken	2009	4, 7.5	Chords, Tones	72	4	yes
Ferguson & Lew‐Williams	2016	7	Tones (with and without communicative prime)	64	12	yes
Ferguson & Waxman	2015	4	Speech, Pictures of dogs, Abstract shapes	40	4	yes
Frank, Slemmer, Marcus, & Johnson	2009	5	Abstract shapes, Speech	96	6	yes
Gerken	2006	9	Speech	48	3	yes
Gerken	2010	9	Speech	36	2	yes
Gerken, Dawson, Chatila, & Tenenbaum	2015	9	Speech	80	4	yes
Gervain & Werker	2013	7	Speech	40	1	yes
Johnson, Fernandes, Frank, Kirkham, Marcus, Rabagliati, & Slemmer	2009	8, 11	Abstract shapes	160	8	yes
Marcus, Fernandes, & Johnson	2007	7	Speech, Tones, Animal sounds, Chords	128	16	yes
Marcus, Vijayan, Bandi Rao, & Vishton	1999	7	Speech	48	3	yes
Pons & Toro	2010	11	Speech	32	2	yes
Rabagliati, Senghas, Johnson, & Marcus	2012	7.5	Sign language‐like gestures	24	2	yes
Saffran, Pollak, Seibel, & Shkolnik	2007	7	Pictures of dogs and cats	44	3	yes
Thiessen	2012	7	Shapes, Tones	128	8	yes
Tsui, Ma, Ho, Chow, & Tseng	2016	9	Faces, Speech	76	5	yes
van Leeuven & Levelt	2016	13	Familiar objects, unfamiliar object, novel objects	59	3	no

### 2.3 Data entry

The first author transcribed the effects of interest, which were double‐checked by both first and last author. We created one database for meta‐analysis, and one for *p*‐curve analysis. The meta‐analysis database contained a fine‐grained subdivision of the included papers into 95 separate records. For each reported experiment, we aimed to record an effect size for each combination of stimulus type (e.g. trained on speech versus trained on tones), age group (e.g. 5‐ and 7‐month‐olds), and training pattern (e.g. trained on ABB versus trained on AAB). These details were not always reported, but some authors did provide them upon request. By contrast, the *p‐*curve database was necessarily less fine‐grained (64 records), as this analysis was only conducted over the key hypotheses tested in each paper (e.g. collapsing together ABB and AAB patterns).

Each record was further coded for a number of factors, including:

- Background information on the report (e.g. title, year, journal, peer‐review status).
- Sample size, mean age of participants (in days), minimum and maximum age, gender ratio, and number of excluded participants.
- Procedure (e.g. headturn preference procedure, central fixation procedure) and familiarization method (e.g. habituation procedure, fixed length familiarization).
- Mean looking times to Novel and Familiar pattern test trials and standard deviations of those times.
- Type of stimuli used (e.g. speech, tones, abstract shapes, etc.).
- Type of training and test patterns (ABB, AAB, ABA).

For subsequent moderator analyses, we coded whether each report used spoken syllables as a stimulus, and whether each report used a stimulus that was communicatively or ecologically meaningful. Our definition of ‘meaningfulness’ was based on its variety of uses in the prior literature (in particular by Ferguson & Lew‐Williams, 2016, and Saffran et al., 2007), and was intended to capture the variety of stimuli that infants might perceive as relevant to their social and/or perceptual experience. Examples of meaningful stimuli include natural categories, such as pictures of dogs (Saffran et al., 2007) or faces (Bulf, Brenna, Valenza, Johnson, & Turati, 2015; Tsui, Ma, Ho, Chow, & Tseng, 2016), and stimuli that have a communicative purpose (e.g. spoken syllables, or the communicatively primed tones in Ferguson & Lew‐Williams, 2016). We dichotomized this predictor for ease of classification and interpretation, but presume that the underlying construct is continuous and multifaceted.

### 2.4 Calculating effect sizes

For each record, we transformed the time that infants attended to novel versus familiar pattern test trials into a Hedges’ *g* statistic (Hedges, 1981). Like Cohen's *d*,* g* is the ratio of the difference between two conditions over the pooled standard deviation, but scaled so that studies with smaller samples are moved closer to 0

$$g\;=\;d(1-\frac{3}{4n-5})$$

where *d* is Cohen's *d* and *n* is the sample size. Positive *g* statistics indicate a preference for Novel pattern trials and negative *g* statistics a preference for Familiar pattern trials.

Some papers did not report means and standard deviations in the text. Although we sometimes could recover these figures from the original authors, we otherwise calculated Hedges’ *g* by deriving Cohen's *d* from reported *t* statistics, using the equation below:

$$d\;=\;t\sqrt{2(1-r)/n}$$

where *r* is the record's by‐subject correlation between responses to novel and familiar trials. Although no studies directly reported *r*, the authors of eight papers (covering 53 records and 561 infants) provided those values for us. For 25 additional records, we could calculate *r* based on the descriptive and inferential statistics provided, using a derivation from Csibra, Hernik, Mascaro, Tatone, and Lengyel (2016). In particular,

$$r\;=\;\left(s_{1}^{2}+s_{2}^{2}-\left[\frac{n{\left(m_{1}-m_{2}\right)}^{2}}{t^{2}}\right]\right)/\left(2s_{1}s_{2}\right)$$

where *m*_i_ and *s*_i_ are the mean and standard deviation of the conditions. For the remaining 17 records, we imputed the by‐subject correlation as the mean *r*, weighted by sample size. The by‐subject correlation was also used to calculate the standard error of each effect size, based on the equation in Cristia (2018); the standard error is used for weighting observations in meta‐regression analyses.

$$SE=\sqrt{\left(\frac{2(1-r)}{n}+\frac{g^{2}}{2n}\right)}$$

## 3 ANALYSES

Our analyses had two goals: To understand what factors moderate infant rule learning abilities, and to assess the strength of the published evidence for these abilities. To understand moderating factors, we used mixed effects meta‐regressions, which also allowed us to estimate the typical size of the learning effect in these experiments. We tested how learning was moderated by the following factors: Whether stimuli were spoken syllables, whether stimuli were communicatively or ecologically meaningful, the age of participants, and the type of pattern used during training. We conducted these analyses using the Metafor package (Viechtbauer, 2010) in R (R Core Team, 2016).

To assess the strength of the published evidence, we employed *p*‐curve analyses (Simonsohn, Nelson, & Simmons, 2014, 2015), which detect publication bias by examining the distribution of statistically significant *p* values between 0 and 0.05. If there is a true effect of learning, then the distribution of *p* values should be right‐skewed with more values closer to 0 than to 0.05. Under the null hypothesis, *p* values are uniformly distributed such that *p* of 0.99 and 0.01 are equally likely. Thus, if published significant findings are solely caused by publication bias (rather than any true effect), then the resulting distribution of *p* values would be uniform (or even left‐skewed if there is so‐called ‘*p* hacking’; Simmons, Nelson, & Simonsohn, 2011). To test for the *presence* of evidential value, a *p*‐curve analysis assesses whether the distribution of *p* values in a sample is more right‐skewed than a uniform distribution; to test for the *absence* of evidential value, a *p‐*curve analysis assesses whether the distribution of *p* values is *less* skewed than would be expected even if all the examined studies had low power (33%) to detect a true effect. These tests are performed by using Stouffer's method to aggregate the probability of obtaining each significant *p* value under the uniform or low power distributions (see Simonsohn et al., 2014, 2015, for further details). Note that Simonsohn et al. (2015) recommend conducting two tests for the presence of evidential value, one on the ‘full’ *p* curve (from 0 to 0.05) and one on the half *p* curve (0 to 0.025), because this procedure is more robust to ambitious attempts to hack the *p* value far below 0.05. For this analysis, the null hypothesis can be rejected if the half *p‐*curve test is significant at *p* < 0.05, or if both *p*‐curve tests are significant at *p* < 0.1.

**Figure 2:**
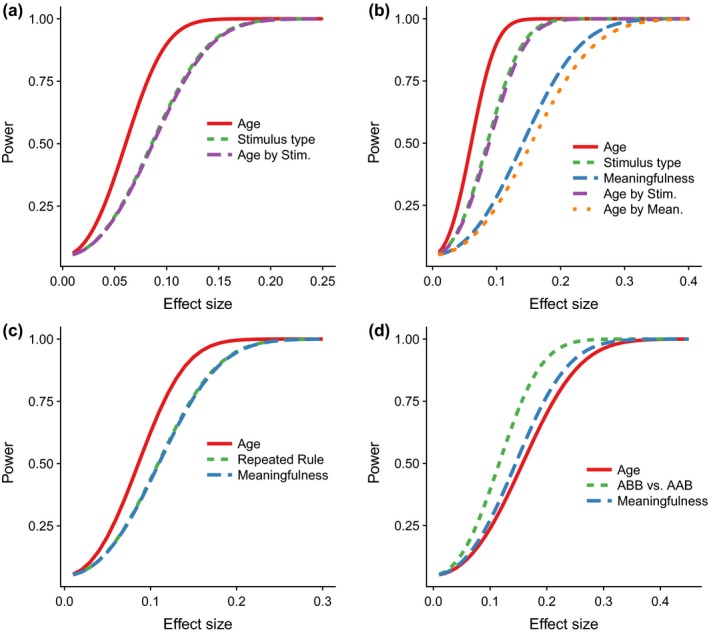
Estimated post‐hoc power across different potential effect sizes for the four meta‐regression analyses reported here. Each line represents a different predictor for the analyses of (A) Effects of stimulus type (speech or not speech) and age; (B) Effects of semantics and age; (C) Comparison of patterns with and without adjacent repetition; (D) Comparison of patterns with early versus late repetition

We caveat that none of our analyses were pre‐registered, nor were power analyses conducted ahead of time. We invite parties interested in reproducing or confirming our findings to explore the data and associated R scripts found at https://osf.io/5k3vw/. Figure 2 displays estimates of post‐hoc power for the set of mixed meta‐regressions reported below (following Hedges & Pigott, 2004). Across our analyses, power was reasonably high; we always had 80% power to detect effects of size 0.25 or greater and, for many moderators, had 80% power to detect effects that were considerably smaller.

## 4 RESULTS

### 4.1 Preliminary analysis: Can infants learn abstract repetition rules?

**Figure 3:**
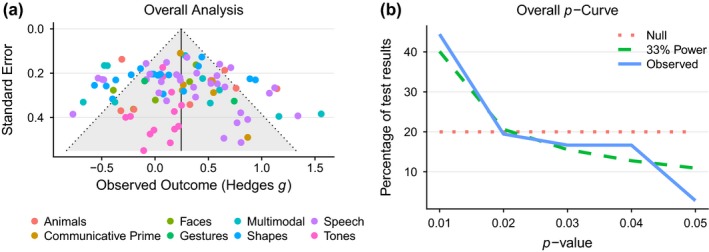
**(**A) Funnel plot of effect sizes against precision (standard error). Shaded region shows 95% confidence interval around the estimated meta‐analytic effect size. Points are colour‐coded according to a coarse breakdown of stimulus type (see colour version of the figure for key). (B) *p*‐Curve plot for all included studies, where the solid blue line shows the observed percentage of significant results in each quintile between 0 and 0.05, the dotted red line shows the expected distribution if there is no true effect of learning, and the dashed green line shows the expected distribution when statistical power is only 33%

The range of effect sizes in our dataset can be seen from the Funnel plot in Figure 3A, plotted against the precision of each estimate, and colour‐coded by the type of stimulus used. A Forest plot, too large to be included here, can also be found at https://osf.io/gx64m/. We estimated the overall effect of learning using a hierarchical random effects meta‐regression with no moderators, and with a random effects structure that modelled how reports were nested in papers which were themselves nested in (i.e. published by) particular labs.

This regression's intercept term (a weighted estimate of the overall effect size) was 0.25, which was significantly different from zero (Standard Error of the estimate = 0.08, *Z* = 3.1, *p* = 0.002, 95% CI = [0.09,0.40]). Thus, averaged across all the experiments, infant learning of abstract repetition rules had a small‐to‐medium effect size. Importantly, however, a test for heterogeneity amongst these effect sizes was also significant (*Q*(94) = 302, *p* < 0.0001), which indicates that moderating variables, such as the stimuli used in the experiments or the age of the participants likely influenced the size of the learning effect.

To assess the evidential value in this dataset, we conducted a *p*‐curve analysis of how the 36 significant *p* values in our dataset were distributed (Figure 3B). The actual distribution of *p* values (blue line) was significantly more right‐skewed than the distribution that would be expected if there were no effect of learning (red line; *p*_full_ and *p*_half_ both < 0.001), indicating significant evidence for the phenomenon that infants can learn abstract repetition rules.

### 4.2 Moderator analysis 1: Effects of speech and age

**Figure 4:**
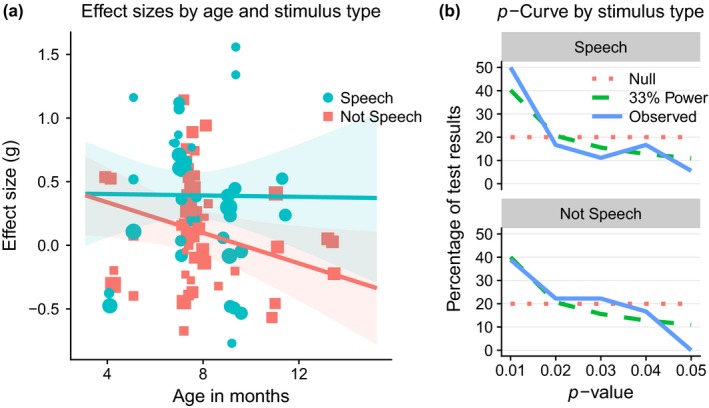
**(**A) Bubble plot showing effect sizes against age, split by stimulus type (speech/not speech), bubble size is inversely proportional to standard error and ribbons show 95% confidence intervals. (B) *p*‐Curves for speech and non‐speech stimuli (see Figure 3B for details)

Next, we assessed whether infants’ learning of abstract repetition rules is facilitated by speech (Marcus et al., 2007). We conducted a mixed effects meta‐regression that included three predictors: a contrast‐coded variable for whether or not the training stimuli were spoken syllables, a scaled and standardized variable for the age of participants (in days), and the interaction of the two. These moderators explained a significant proportion of the variance (QM(3) = 14.2, *p* = 0.003); the results of the regression are displayed in Table 2 and Figure 4A. This meta‐regression analysis confirmed Marcus and colleagues’ observation that infants were better able to learn abstract repetition rules from spoken syllables than other stimuli (estimated increase in Effect size = 0.20, *SE* = 0.062, *z* = 3.2, *p* = 0.001).

**Table 2:** Results of a meta‐regression using stimulus type, age and their interaction as moderators

	β (Standard Error)	*z*	*p*	95% CI
Intercept	0.25 (0.082)	3.1	0.002	[0.09,0.41]
Age	−0.059 (0.054)	−1.1	0.27	[−0.16,0.05]
Stimulus type (Speech)	0.20 (0.062)	3.2	0.0013	[0.08,0.32]
Age* Stimulus interaction	0.076 (0.056)	1.3	0.18	[−0.04,0.19]

This same analysis also allowed us to test Dawson and Gerken's (2009) proposal that infants’ learning abilities narrow with age, such that younger infants learn abstract repetition rules from a variety of stimuli but older infants are focused on speech. That proposal predicts a significant interaction between age and stimulus type, such that the effect size for non‐spoken stimuli declines with age but the effect size for spoken stimuli does not. However, while the interaction was in the predicted direction (Table 2 and Figure 4A), it was not statistically significant (β = 0.08, *SE* = 0.06, *z* = 1.3, *p* = 0.18). Perhaps surprisingly, the coefficient for the age predictor suggested that the ability to learn abstract repetition rules declined with age irrespective of stimuli, although importantly this trend was not statistically significant (β = −0.06, *SE* = 0.05, *z* = −1.1, *p* = 0.27).

A *p*‐curve analysis shed light on the evidential strength for the speech advantage (Figure 4B). The studies that show statistically significant learning from speech (*n* = 18) provided significant evidential value (*p*_full_ and *p*_half_ both < 0.0001). Interestingly, however, the studies that showed statistically significant learning from non‐speech (also *n* = 18) also provided significant evidential value (*p*_full_ < 0.0001 and *p*_half_ = 0.0035), despite the estimated effect size being smaller when infants learned from non‐speech. This suggests that infants can also learn abstract repetition rules from some (but perhaps not all) non‐speech stimuli. And consistent with the possibility that infants learn from some but not all non‐speech stimuli, the meta‐regression also had significant residual heterogeneity (*Q*(91) = 297, *p* < 0.0001), indicating that factors other than speech and age are likely to have affected learning in these tasks.

Thus, the meta‐regression analysis suggested that speech does indeed facilitate learning of abstract repetition rules. However, the *p‐*curve analysis suggested that a speech bias may not be a sufficient explanation of infants’ learning abilities, by showing that there is significant evidence that infants can learn abstract repetition rules from non‐speech stimuli.

### 4.3 Moderator analysis 2: Meaningful stimuli and age

Might the facilitative effect of speech reflect a broader tendency to learn abstract repetition rules from any communicative and meaningful stimulus (Ferguson & Lew‐Williams, 2016; Saffran et al., 2007)? We used meta‐regression to test whether stimulus type (speech/non‐speech) predicted effect size above‐and‐beyond any effect of communicative or ecological meaningfulness. To do this, we included a contrast coded predictor for meaningfulness, a predictor for age, and their interaction, as well as a predictor for stimulus type that was first residualized against the predictor for meaningfulness, along with an interaction between residualized stimulus type and age. This regression thus tested if the speech/non‐speech distinction explained behaviour that was *not* already explained by meaningfulness (see Baayen, Feldman, & Schreuder, 2006, for a related approach). Again, moderators explained a significant proportion of the variance (QM(5) = 19.6, *p* = 0.002).

**Figure 5:**
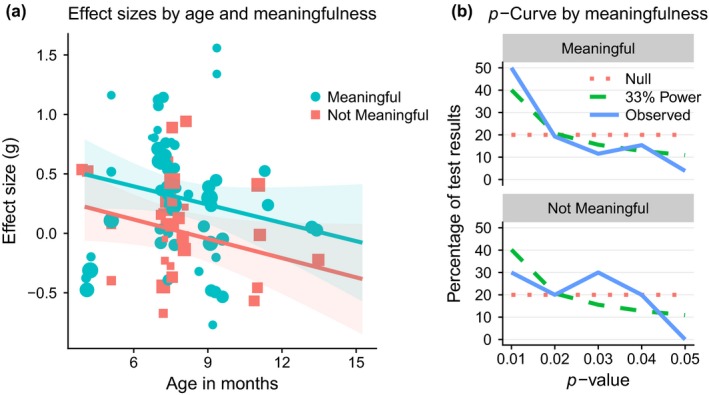
**(**A) Bubble plot of effect size against age, split by stimulus type (meaningful/not meaningful). (B) *p‐*Curves for meaningful and not meaningful stimuli

In this meta‐regression, the meaningfulness of the stimulus significantly predicted the size of the learning effect (β = 0.22, *SE* = 0.06, *z* = 3.9, *p* < 0.0001, see Table 3 and Figure 5A): Infants were better able to learn repetition rules from meaningful stimuli, and showed a lower effect size when learning from meaningless stimuli such as geometric shapes or tones. Interestingly, the predictor for whether or not stimuli were speech did not significantly explain any additional variance in the effect size above‐and‐beyond the effect of meaningfulness (β = 0.13, *SE* = 0.11, *z* = 1.2, *p* = 0.22).^2^

**Table 3:** Results of a meta‐regression in which moderators are stimulus meaningfulness, age, residualized stimulus type, and the interactions between stimulus type and age, and meaningfulness and age

	β (Standard Error)	*z*	*p*	95% CI
Intercept	0.16 (0.081)	2.0	0.047	[0.002,0.32]
Age	−0.089 (0.05)	−1.8	0.077	[−0.19,0.01]
Stimulus type (Speech, residualized)	0.13 (0.11)	1.20	0.22	[−0.08,0.34]
Meaningfulness (Meaningful)	0.22 (0.06)	3.9	<0.0001	[0.11,0.33]
Age * Stimulus type interaction	0.089 (0.085)	1.00	0.30	[−0.078,0.26]
Age * Meaningfulness interaction	0.037 (0.05)	0.73	.47	[−0.062,0.14]

There were no significant interactions with age, whether against meaningfulness or stimulus type. The estimated effect size declined with age in a statistically marginal fashion (β = −0.09, *SE* = 0.05, *z* = −1.8, *p* = 0.08); such a decline might be expected if researchers tested older infants on harder tasks. Additionally, the test for residual heterogeneity in the regression was still significant (*Q*(89) = 290, *p* < 0.001), suggesting that further explanatory factors remain to be discovered.

A *p*‐curve analysis indicated that there was strong evidence for the claim that infants learned abstract repetition rules in the 26 significant experiments that used meaningful stimuli (*p*_full_ and *p*_half_ both < 0.001, Figure 5B), that is, the *p* curve was more right‐skewed than the uniform. But interestingly, there was not significant evidence that infants also learned abstract repetition rules in the 10 significant experiments that used meaningless stimuli (*p*_full_ = 0.01 and *p*_half_ = 0.11; recall that the test needs to be significant at *p* < 0.1 for both measures); that is, the *p* curve was not significantly more right‐skewed than the uniform. We followed up this null result by testing if the *p* curve contained significantly less evidence than a simulated ‘low power’ *p* curve (i.e. is significantly less right‐skewed than the green dashed line in Figure 5B); however, this test was also not significant (*p*_full_ = 0.31 and *p*_half_ = 0.57). We thus conclude that the current evidence does not support conclusive claims as to whether or not infants can learn abstract repetition rules from meaningless stimuli. By contrast, it seems clear that infants do easily learn these rules from meaningful stimuli, and the combination of the meta‐analysis and *p‐*curve analysis suggests that infants are significantly better at learning these rules from meaningful stimuli than from non‐meaningful stimuli.

### 4.4 Moderator analysis 3: Effect of pattern type

Is it easier for infants to learn some abstract repetition patterns than others? For example, there is some evidence that infants find reduplicated patterns (ABB, AAB) easier to learn than non‐reduplicated patterns (ABA, Johnson et al., 2009), and find edge‐final reduplications (ABB) easier to learn than edge‐initial reduplications (AAB; Rabagliati et al., 2012). We used meta‐regression to test whether the size of the learning effect varied based on which pattern infants were trained on. This analysis used a subset of the entries in our database for which the training pattern could be coded (71 entries total).

We conducted two meta‐regression analyses. First, we tested whether the learning effect size was greater for reduplicated patterns like AAB and ABB (48 entries), than for ABA patterns (23 entries). We included infant age and stimulus meaningfulness as control predictors. However, reduplication was not a significant predictor (β = 0.06, *SE* = 0.05, *z* = 1.13, *p* = 0.26), that is, our regression did not suggest that ABA patterns were harder to learn than ABB or AAB.

A second meta‐regression tested whether ABB patterns (31 entries) were easier to learn than AAB (17 entries) patterns; we again included age and meaningfulness as control predictors. The effect size for ABB patterns was indeed larger than the effect size for AAB patterns (β = 0.28, *SE* = 0.09, *z* = 3.0, *p* = 0.003), a finding that is consistent with prior conclusions that infant pattern learning is facilitated by a recency effect (Johnson et al., 2009; Rabagliati et al., 2012).

## 5 DISCUSSION

These analyses permit three strong conclusions. First, while the literature on how infants learn abstract repetition rules may be somewhat confusing, it still provides strong evidence that infants can learn these abstract patterns. Across a variety of stimuli, from speech to shapes, infants demonstrated a small but reliable effect of learning, that a *p*‐curve analysis indicated could not be explained away as publication bias. Second, we can conclude that infants learn these abstract patterns more reliably from spoken syllables than from a variety of other stimuli: learning from syllables increased the size of the learning effect by *g* = 0.21. Finally, our meta‐regression provided evidence that the advantage for spoken stimuli is in fact more parsimoniously explained as an advantage for learning from any type of stimulus that is meaningful to the infant, for example, through its communicative value or ecological familiarity. Once our meta‐regression analyses accounted for meaningfulness, the additional effect of speech on learning was not significant.

Our analyses were more equivocal about three further points. First, while there was some indication that the effect of learning was smaller in older infants (e.g. in the second moderation analysis), there was no significant evidence for marked changes in learning abilities with age (cf. Dawson & Gerken, 2009) despite the statistical power of this meta‐analysis being quite high. Second, it remains unclear whether or not infants can learn abstract patterns from stimuli that are not meaningful, such as abstract shapes. Our meta‐regressions indicated that it is *harder* for infants to learn repetition rules from these stimuli, but the *p*‐curve analysis did not rule out that such rules are learnable from these stimuli. Finally, we provided limited evidence that some repetition rules are easier to learn than others. Contra prior claims, we did not find evidence that patterns containing immediate reduplication (ABB/AAB) are easier than non‐reduplication patterns (ABA) despite high statistical power, but there was some evidence for a recency effect in learning: ABB patterns were easier to learn than AAB patterns. We return to this point in the general discussion.

## 6 EXPERIMENT

**Figure 6:**
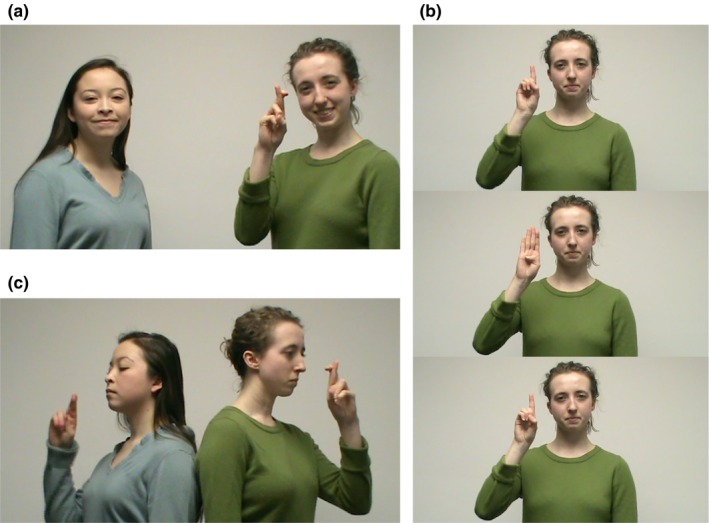
(a) Still frame from the Communicative pre‐exposure video in the multi‐lab experiment. (b) Still frame from the Non‐Communicative pre‐exposure video in the multi‐lab experiment. (c) Sequence of still frames from a video used in the habituation phase (in this case, the gesturer produces an ABA pattern). Test phase videos were similar to this

The key finding from the meta‐analysis was that infants’ learning of abstract repetition rules was enhanced by stimuli that are meaningful, including but not limited to speech. To complement the meta‐analysis, we conducted a cross‐lab experiment, which aimed to confirm this claim. This experiment was an extension of two previous publications: Rabagliati et al. (2012) and Ferguson and Lew‐Williams (2016). The former is important because it is perhaps the only study to argue that abstract rule learning in infancy is better explained by enhanced learning from spoken stimuli than by enhanced learning from meaningful stimuli. They found that 7.5‐month‐old infants learned ABB patterns from sequences of sign language‐like gestures (similar to those in Figure 6c), but failed to learn AAB patterns, a pattern of results that strikingly echoed infants’ behaviour when learning patterns from abstract shapes (Johnson et al., 2009). From these data, they argued against an advantage for meaningful stimuli because, while the sign language‐like gestures (hereafter simply referred to as gestures) had communicative features, infants could not learn patterns as robustly from these stimuli as they could from speech.

However, this conclusion is at odds with both our meta‐analysis and subsequent experiments, in particular Ferguson and Lew‐Williams’ (2016) finding that infants will learn abstract repetition rules from pure tones when those tones are primed to be communicative. Furthermore, it is not clear whether Rabagliati et al.'s gestures were sufficiently communicative and meaningful as to elicit learning, as those properties were only cued by eye contact with a videotaped gesturer.

We drew on this point in a three‐condition experiment. In a control condition, 7.5‐month‐old infants participated in a study similar to Rabagliati et al. (2012). We compared their performance to infants in a second condition who, following Ferguson and Lew‐Williams (2016), were specifically primed to consider gestures as communicative and meaningful, as well as to infants in a third condition who were primed to consider gestures as *non*‐communicative. To prime infants’ interpretations, they viewed a short video of two people using gestures communicatively or non‐communicatively; the control group did not watch a pre‐exposure video. Then, infants were habituated to silent videos of one person producing ABB or ABA sequences of novel hand gestures. Finally, infants viewed videos of novel gesture sequences that either matched the pattern from the familiarization sequences (Familiar trials) or followed a different pattern (Novel trials).

One noteworthy aspect of this experiment is that half of the data in each condition were collected in North America and half in Europe, allowing us to compare consistency in results across laboratories.

### 6.1 Method

#### 6.1.1 Participants

Sixty‐three monolingual English‐learning, full‐term, typically developing 7‐month‐old infants with no known history of learning or language impairments were tested (*M* = 7.28 months; range = 7.00–8.00; 33 females). Infants were randomly assigned to each of the ‘Communicative’ (*n* = 21), ‘Non‐Communicative’ (*n* = 21), or ‘No Exposure’ (*n* = 21) conditions, respectively; within each condition, they were also randomly assigned to be habituated to either an ABB or ABA pattern. Following identical exclusion criteria from prior pattern‐learning experiments (Ferguson & Lew‐Williams, 2016), additional infants were tested but excluded for fussing or crying (*n* = 11), providing mean log‐transformed looking times that were more than 2.5 standard deviations from the condition mean (*n* = 2), failing to contribute at least two familiar and two novel trials at test (*n* = 20), parental interference (*n* = 3), or technical error (*n* = 2). Informed consent was obtained for each participant. All procedures were approved by the human subjects committees at Princeton University and the University of Edinburgh. All stimuli and procedures were approved by the Institutional Review Board at Princeton University, following US Federal Policy for the Protection of Human Subjects, and the Psychology Research Ethics Committee at the University of Edinburgh, following British Psychological Society standards.

#### 6.1.2 Stimuli

In each of the experiment's three phases—pre‐exposure, habituation, and test—infants observed pre‐recorded videos in which one or more actors produced gestures based on American Sign Language.^3^

During a pre‐exposure phase, infants in the Communicative and Non‐Communicative conditions observed one of two movies. Both videos included two female actors producing sequences of gestures using six distinct ASL handshapes: *C*,* Flat‐O*,* Q*,* R*,* X*, and *Y*. In the Communicative condition, one actor used these gestures to communicate with the other actor and the infant, and the other actor responded using speech; this interaction thus carried a number of communicative hallmarks such as turn taking, speech, and eye contact. In the Non‐Communicative condition, both actors synchronously produced the same sequences of gestures while oriented away from each other and the infant; this interaction was thus a joint activity, but it lacked the hallmarks of communication and interaction listed above. Because these communicative hallmarks were combined, we cannot determine whether one or more of the hallmarks might be key to infant learning, but the combination of cues provides a rich and inclusive test of whether cues to meaningfulness influence learning.

Note that no patterns were taught in these primes. Figure 6 shows stills from the videos, while scripts can be found at https://osf.io/5k3vw/.

Next, during the habituation phase, infants observed videos in which tokens from a different set of ASL gestures were arranged to form three‐token sequences conforming either to an ABB or ABA pattern, depending on habituation condition (Figure 6c). In each video, the actor produced sequences combining a random A token (from *Bent‐Five*,* U*,* 1‐I*,* L*) with a random B token (from *O*,* L‐I*,* B*, and *Six)* in either ABB or ABA form. Each entire sequence lasted approximately 5 seconds, with 1.33s for each gesture and approximately 0.5s of neutral standing before and after.

Finally, during the test phase, a different set of A (*Five*,* V*) and B (*H*,* I*) gestures were combined to form four new ABB and ABA sequences. These had the same timing properties as in habituation, and were produced by the same actor.

In all videos, gestures were produced with similar timing: the actor took approximately 0.66s to raise her hand from a neutral position and form the sign, and another 0.66s to lower her hand back to the neutral position. Habituation and test gestures were produced above the right shoulder, allowing infants to clearly see the sign shape without blocking the actor's face.

#### 6.1.3 Procedure

Infants in the Communicative and Non‐Communicative conditions began the study with a pre‐exposure video. Infants assigned to the No Exposure condition proceeded immediately to habituation.

The habituation phase consisted of up to 25 trials in which infants observed videos of three‐gesture sequences that conformed to either an ABB or an ABA pattern. On each trial, the 16 possible sequences were shuffled and then played in a repeating block until either the infant looked away for 2 consecutive seconds or the trial reached the maximum length of 120 seconds. The phase ended when infants’ cumulative looking time to any three consecutive trials was less than 50% of their total looking time to their first three trials, or until they reached the maximum of 25 habituation trials (*n* = 5).

In the test phase, infants observed new gestures arranged to follow both ABB and ABA patterns in a series of eight trials. Trials were arranged into two blocks of four; each block contained two ABB and two ABA trials. Each trial contained four distinct sequences, shuffled and repeated; the parameters were otherwise the same as in test trials.

#### 6.1.4 Data preparation

As in Rabagliati et al. (2012), we excluded test trials in which infants did not look long enough to recognize whether a gesture sequence was ABA or ABB (i.e. less than 3.32s, the time from video onset to display of the third handshape). The final sample contributed on average 3.6 novel trials (*SD* = 0.7) and 3.6 familiar trials (*SD* = 0.6). We then log‐transformed looking times to account for positive skew (Csibra et al., 2016).

To assess learning, we subtracted mean log‐transformed familiar trial looking time from mean log‐transformed novel trial looking time; a positive value thus indicates a novelty preference.

#### 6.1.5 Predictions

Our primary prediction was that infants’ learning would vary by condition, with infants showing stronger evidence of learning (i.e. a stronger novelty preference) if they had been exposed to gestures as a communicative signal, but not if they had witnessed non‐communicative actions, or had no exposure at all.

### 6.2 Results

**Figure 7:**
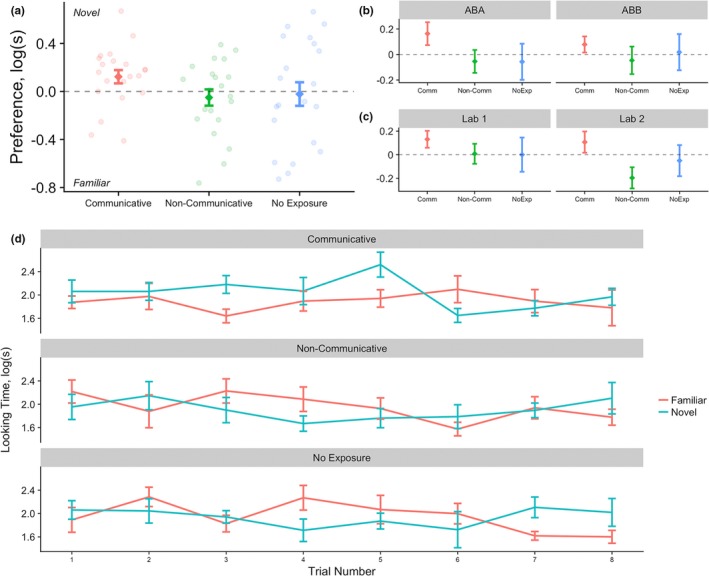
Infants’ mean log‐transformed novelty preferences by (A) condition, (B) habituated pattern, (C) lab in which they were tested. (D) Log‐transformed looking times by condition and test trial number. Error bars indicate ±1 *SE*. Semi‐transparent points indicate by‐participant individual observations

Overall, we found no significant difference in infants’ looking to novel and familiar trials at test (*M*_diff‐log_ = 0.017 [95% CI = −.07,0.11], *t*(62) = 0.39, *p* = 0.70), and no significant effect of condition on test preferences (*F*(2, 60) = 1.50; *p* = 0.23). Nevertheless, planned comparisons (Figure 7A) within each condition showed that infants in the Communicative condition showed a significant preference for novel trials at test (*M*_diff‐log_ = 0.12 [.03,0.23], *t*(20) = 2.23, *p* = 0.037, *d* = 0.49), but infants in the Non‐Communicative condition did not show a significant preference (*M*_diff‐log_ = −0.050 [−0.19,0.08], *t*(20) = −0.74, *p* = 0.47, *d* = −0.16). In the No Exposure control, the infants also did not show a significant preference (*M*_diff‐log_ = −0.021 [−0.21,0.16], *t*(20) = −0.21, *p* = 0.83, *d* = −0.05); moreover, unlike in Rabagliati et al. (2012), infants in this control group did not show evidence of learning ABB patterns (see Figure 7B). Non‐parametric Wilcox and Binomial tests yielded a converging pattern, with infants in the Communicative condition showing a significant novelty preference (Wilcox *p* = 0.035, binomial *p* = 0.027) but infants in the Non‐Communicative and No Exposure conditions not showing significant differences between novel and familiar looking times.

A second, hierarchical model suggested that this pattern across conditions correlated with a difference in the way infants allocated their attention to the test trials over the test phase. This hierarchical model predicted log‐transformed trial looking times using fixed effects of Trial Number (1–8), Trial Type (novel, familiar), and Condition (Communicative, Non‐Communicative, and No Exposure; contrast‐coded with Communicative as reference level) and random by‐subject intercepts and by‐subject slopes of Trial Number and Trial Type. This model yielded a significant effect of Trial Number (β = −0.027, *SE* = 0.012, χ^2^ = 4.82, *p* = 0.028) as well as a significant three‐way interaction between Trial Number, Trial Type, and Condition (β = −0.12, *SE* = 0.046, χ^2^ = 6.52, *p* = 0.011). Examining the time course of infant looking times during test (see Figure 7D) showed that this interaction was driven by an early preference for novel trials in the Communicative condition which faded over trials as infants’ attention waned.

Building on this approach, we examined the effect of Habituated Pattern (ABB, ABA) and Lab (Lab 1, Lab 2) on infants’ looking times by adding them as additional factors to the hierarchical model above. Neither had any significant effect either independently or in interaction with other factors; infants’ behaviour did not significantly vary based on the pattern to which they were habituated or the lab in which they were tested (see Figure 7B and C), although it is unclear whether these findings are true null effects, or reflect lack of statistical power.

There was no significant difference in infants’ looking during habituation, with infants across conditions looking on average for 114.4s to the gesture sequences over 9.8 trials.

### 6.3 Discussion

This experiment was designed to test two previous claims: that infants only show limited success at learning abstract repetition rules from sign language‐like gestures, and that infants are more likely to learn such rules from stimuli that are primed as communicative and meaningful. When infants were trained on gestures without a prior exposure period, they showed no significant evidence for learning. However, after infants viewed a video that was designed to prime gestures as communicative and meaningful, we did observe significant learning, consistent with both Ferguson and Lew‐Williams (2016) and the conclusions of our meta‐analysis. Moreover, we observed no evidence of learning in infants who were shown a similar prime video, in which gestures were not treated as communicative.

These results thus broadly converge with the claim that meaningfulness affects infants’ success in learning abstract repetition rules. It remains possible that our pattern of results could be driven by a confluence of other factors (for example, the non‐communicative prime may confuse infants and inhibit their learning, while infants in the no exposure condition may be more attentive to the actors’ faces than their hands), but we suggest that only meaningfulness offers a parsimonious account of why infants behaved as they did across the conditions of this study. In addition, the results of this study have implications for the interpretation of Rabagliati et al. (2012); they found significant evidence that infants learned ABB patterns from unprimed gestures, but we found no significant evidence of this. This raises the possibility that Rabagliati and colleagues may have over‐estimated infants’ learning abilities in this case.

However, we should note that the interpretation of this experiment has important statistical qualifications. In particular, while infants in the Communicative condition showed statistically significant learning in our main analysis, and infants in the other conditions did not, there was no significant overall effect of condition, that is, infants were not significantly better at learning abstract repetition patterns from the stimuli that were primed to be meaningful than from the other stimuli. Instead, a significant difference between conditions was only found in a follow‐up analysis that accounted for the time‐course of the experiment's test phase. Despite this, we still suggest that readers can be reasonably confident in drawing conclusions from this study, because the key finding—significant learning from a meaningful stimulus—converges well with the results of the meta‐analysis. This illustrates the advantages of our combined meta‐analytic and experimental approaches.

## 7 GENERAL DISCUSSION

Using both meta‐analytic and experimental methods, we assessed abstract rule learning in infancy and its domain specificity. Contra concerns about replicability in infant research (e.g. Frank et al., 2017), we found statistically robust evidence that infants can learn abstract repetition rules, and confirmed prior reports (Marcus et al., 2007) that it is easier to learn such rules from speech than a variety of other stimuli. But both our meta‐analysis and experiment also indicated that a domain‐specific advantage for learning from speech is not a parsimonious account. Rather, our review of prior studies and our own experiment showed that the strongest evidence for learning repetition rules came when experiments used stimuli that were communicatively and ecologically meaningful, and that this factor accounted for the advantage for learning from speech.

This work provides the most comprehensive profile of infant abstract rule learning to date, with the meta‐analysis alone incorporating data from 20 journal articles and reports, 63 separate experiments, and 1,318 infants. Amidst concerns about replicability, our results present a more positive picture. Both our meta‐analyses and our *p‐*curve analyses confirmed the basic phenomenon that infants can learn abstract repetition rules, supported many of the literature's claims, and indicated that the most important results could not be explained by publication bias. However, given conflicting findings in the rule learning literature, not every claim could be supported. For instance, we did not find definitive evidence that infants’ learning abilities undergo a developmental specialization, such that younger infants are able to learn repetition rules from a wider array of stimuli (Dawson & Gerken, 2009). We also found little evidence that infants can learn repetition rules from abstract, meaningless stimuli, such as shapes or tones. Although such learning has been reported in the literature (e.g. Dawson & Gerken, 2009; Johnson et al., 2009; Thiessen, 2012), a *p*‐curve analysis suggested that, when aggregated, these findings did not contain statistically significant evidential value. This null finding can be explained in multiple ways. In part, it is likely indicative of publication bias, but we would argue that it also indicates that previously reported studies had low statistical power (learning from abstract, meaningless stimuli is likely to be a small effect), and also could reflect low statistical power in that particular *p*‐curve analysis (which only examined 10 significant findings).

In our experiment, normal‐hearing infants provided evidence of learning abstract repetition rules from a novel signal—sign language‐like gestures—if they first witnessed the signal being used in a conversational, turn‐taking exchange between two people. Akin to Ferguson and Lew‐Williams (2016), we argue that this effect is not reducible to general social attention, as infants did not provide evidence of learning when the prime showed two people producing gestures in a similarly interesting but distinctly non‐communicative fashion, or when infants received no prior exposure to gestures. We argue that the suite of communicative cues in the prime caused infants to treat the gestured stimuli as meaningful, and that this facilitated their extraction of repetition rules, consistent with the main conclusion of the meta‐analysis.

The experiment also illustrates three further points. First, and as mentioned before, any effect of learning abstract repetition rules from stimuli that are not meaningful is likely to be extremely small, as evidenced by the lack of learning after the non‐communicative prime and in the no exposure condition. Second, it is easier to draw inferences from experiments conducted in combination with a quantitative analysis of the prior literature. In particular, while we did not find significant differences between the three conditions of our experiment (i.e. interaction effects), the pattern of results was still consistent with the findings from our meta‐analysis, leading us to have more confidence in our conclusion than would be warranted by the results of the experiment alone. Finally, our experiment leads to a preliminary suggestion that the data from studies of repetition rule learning may be consistent enough across laboratories to enable jointly conducted experiments. As Figure 7C shows, some of the key patterns in our data (particularly successful learning from stimuli primed to be meaningful) were roughly similar in both the European and American samples, suggesting that combining data across sites is a viable strategy for increasing sample size without overly diluting the signal‐to‐noise ratio.

The conclusion that learning of abstract repetition rules is not simply specific to speech echoes prior findings about putative mechanisms of language acquisition (Marcus & Rabagliati, 2012). Categorical perception, for example, was argued to be a domain‐specific adaptation for language, until further work showed that it was neither specific to speech (e.g. Beale & Keil, 1995; Burns & Ward, 1978) nor to humans (Kuhl & Miller, 1978). Similarly, fast mapping was initially assumed to apply to word learning (Carey & Bartlett, 1978), but has since been shown to apply across domains (Coutanche & Thompson‐Schill, 2015; Markson & Bloom, 1997). Importantly, however, while our results suggest that rule learning is not specific to speech, they do not suggest that infants can learn repetition rules from just any stimulus, because infants appear to have difficulty learning rules from arbitrary stimuli such as geometric shapes or tones. Interestingly, this suggests that the ability to learn an abstract repetition rule may still dissociate from a skill such as statistical learning of transition probabilities, which does appear to operate over such stimuli (Kirkham, Slemmer, & Johnson, 2002; Saffran, Aslin, & Newport, 1996; Saffran, Johnson, Aslin, & Newport, 1999).^4^

We thus suggest that infants are more likely to learn abstract repetition rules from domains of stimuli that are delineated by whether they are communicatively or ecologically meaningful to the infant. This concept of meaningfulness is purposely quite broad; it suggests that infants should be able to learn abstract rules from stimuli whose meaningfulness derives from the infants’ day‐to‐day experiences with sounds, objects, and people (cf. Saffran et al.'s 2007 suggestion that infants learn from stimuli that they can classify), as well as from stimuli that are accompanied by strong cues to their relevance. Such cues might include integration of the stimulus into a conversation, as well as pedagogical cues such as eye contact or body posture (see Ferguson & Waxman, 2017, for discussion). We argue that while the concept of meaningfulness can certainly be refined further, it currently serves as a more powerful explanation than potential alternative accounts, such as accounts based on the psychophysical properties of the stimuli. For example, one account suggested to us by an anonymous reviewer is that learning may be easier from perceptually complex stimuli, like speech or animal pictures, compared to simpler stimuli, like tones or shapes. This perceptual account is potentially problematic because it cannot explain why a communicative (vs. non‐communicative) prime might change the learnability of a stimulus, as found in our experiment using handshapes and in Ferguson and Lew‐Williams (2016) using tones.

Another alternative, suggested by a different anonymous reviewer, is that the present data patterns might be better explained by processing difficulty than meaningfulness, that is, infants learn better from stimuli that they find easier to process. While the concept of processing difficulty will surely be important for theories of how abstract repetition rules are learned (a point that is potentially illustrated by our meta‐analytic finding that infants learn ABB patterns more easily than AAB patterns), it is at the same time unclear how to define what should make a stimulus difficult for an infant to process. For example, spoken syllables could be considered either easy to process (because they are frequent in the infants’ environment) or difficult to process (because they are acoustically and perceptually complex); and pure tones could be considered either difficult to process (because they are infrequent) or easy to process (because they are acoustically and perceptually simple). Without an independent metric for what makes a stimulus difficult to process during the act of pattern learning, theories based on processing difficulty will be unable to make precise predictions about what stimuli infants should, and should not, have difficulty learning from. Therefore, we suggest that this concept does not, at present, provide a satisfying explanation of infants’ successes and failures at learning abstract repetition rules. However, this discussion also highlights a potential difficulty with the construct of meaningfulness: What makes a stimulus meaningful is in the eye of the beholder, and is thus not directly observable. But approximations to meaningfulness can still be measured in terms of infants’ stimulus preferences or everyday experiences with objects, making the construct more testable. The potential importance of meaningfulness for pattern learning is consistent with an array of other work on how communicative and pedagogical cues might influence learning in even very young infants (Csibra & Gergely, 2009; Ferguson & Waxman, 2016; Senju & Csibra, 2008). However, it remains to be seen whether learning of abstract patterns is enhanced by meaningful stimuli because these stimuli recruit domain‐specific learning mechanisms (cf. natural pedagogy theory; Csibra & Gergely, 2009), or because these stimuli demand fuller engagement of more general‐purpose learning algorithms.

Away from questions of domain specificity, our meta‐analysis also has implications for the mechanisms by which infants create abstract generalizations. Marcus et al.'s (1999) original proposal was that infants extract rules based on symbolic variables from the input, but others have suggested that infants’ behaviour in repetition rule tasks may instead be driven by a simpler perceptual mechanism that allows them to detect adjacent repetitions, based on evidence that newborns learn ABB patterns but not ABA patterns (Gervain et al., 2008; Mehler, Nespor, & Peña, 2008). The repetition‐detector proposal predicts that infants should show stronger evidence of learning from patterns containing adjacent repetitions (i.e. ABB/AAB vs. ABA; cf. Johnson et al., 2009), but our meta‐analysis did not support this: the estimated difference in effect size for adjacent versus non‐adjacent rules was only 0.05. Our results are thus more consistent with Marcus’ suggestion, although it remains possible that the suggested perceptual primitive may better characterize learning in younger infants.

Although this meta‐analysis provides a particularly broad picture of how infants learn abstract repetition rules, we see three potential concerns that might be raised about it. The first concern is whether a meta‐analysis like this permits strong causal claims. Meta‐analysis alone cannot show that a factor such as stimulus meaningfulness actually causes easier learning, because the conditions for causal inference, such as random assignment, have not been met; this is an important reason to augment meta‐analyses with complementary experiments. We would thus suggest caution in interpreting some ancillary results of our meta‐analysis, such as our finding that infants were better able to learn ABB than AAB patterns. While this finding is not unexpected given the literature, and while we controlled for important confounding variables in our analysis (e.g. participant age, stimulus meaningfulness), certain selection effects could have biased this result (e.g. perhaps researchers used differentially learnable stimuli to test ABB versus AAB patterns). The second concern is whether the results of the meta‐analysis—which focused on behavioural studies of how children learn simple repetition rules—might generalize to other experimental paradigms, such as those that rely on neuroimaging (e.g. Gervain et al., 2008), or to other examples of learning patterns and rules (such as learning of phonological or morphosyntactic rules; e.g. Gomez & Lakusta, 2004; Seidl & Buckley, 2005). The degree of generalization is currently unclear.

The third and most important concern, with broad methodological and theoretical implications, is how meta‐analyses like this should account for directional preferences in infant looking time tasks. While most studies in our dataset reported novelty preferences, there is also a subset that instead reported a preference for familiar stimuli. It is well known in infant research that the type of preference observed in an individual study is not easy to interpret (see e.g. Oakes, 2010, for discussion), but how should we respond when preferences differ between studies?

One possibility is that subtle moderating factors, such as participant age, might drive different preferences; our meta‐analysis probably lacks the power to disentangle these factors from other small differences between studies. But there is also another possibility, that these familiarity preferences are not in fact indicative of true effects but are instead statistically inevitable Type I errors, or what Gelman and Carlin (2014) call Type S (for sign) errors.

This logic runs as follows. If we assume that experiments on learning repetition rules should probabilistically elicit a novelty preference, then infants who have learned a repetition rule should very rarely show a familiarity preference. However, infants who have *not* learned a rule should be equally likely to show a false positive familiarity preference as a false positive novelty preference. Given this, a significant familiarity preference is more likely when infants have *not* learned a repetition rule than when they have learned a rule. We used *p*‐curve analyses to assess this idea, comparing the evidence from the 28 experiments that reported a novelty preference and the eight experiments that reported a familiarity preference. We reasoned that if the two types of preference are equivalent, then they should provide similar amounts of evidence. But, if it is the case that learning repetition rules cause a novelty preference, then studies that report novelty preferences should contain significant amounts of evidence (i.e. right‐skewed *p* curves), while studies that report familiarity preferences should contain little evidence (i.e. flat or left‐skewed *p* curves).

**Figure 8:**
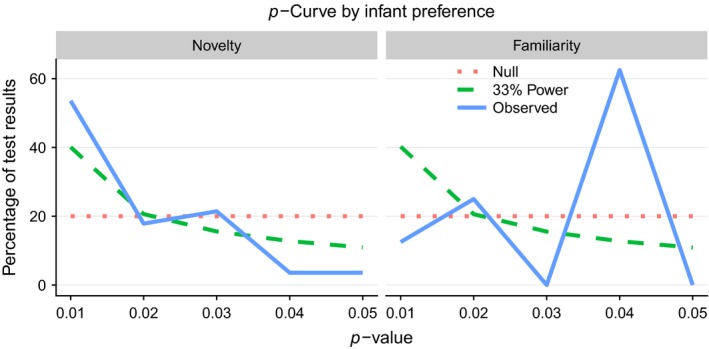
*p‐*Curve analyses for studies in our dataset split by whether they showed a novelty or familiarity preference. See Figure 3B for details

As Figure 8 shows, the *p*‐curve results conformed to the second possibility. The set of studies in which infants showed a novelty preference contained significant evidential value (*p*_full_ and *p*_half_ both < 0.0001), but the set of studies in which infants showed a familiarity preference did not contain significant evidential value (*p*_full_ = 0.60 and *p*_half_ = 0.53). In fact, the studies showing a familiarity preference provided significantly less evidential value than would be expected if those studies had only had 33% power to detect an effect (*p*_full_ = 0.05 and *p*_half_ = 0.07; recall that this test is significant if both measures are *p* < 0.1). In addition, the evidential value for the studies showing a familiarity preference appeared to be lower than the evidential value for the studies showing a novelty preference: Based on the *p‐*curve analysis, we estimated that power for the studies showing a familiarity preference was only 5%, with a 95% confidence interval of 5%–34%, and this did not overlap with the confidence interval for those studies showing a novelty preference (*M* = 82% with 95% CI of 67%–92%). These analyses thus suggest that inevitable publication bias may explain the occasional appearance of familiarity preferences in studies of repetition rule learning.

This finding, that only the dominant infant response (here, a novelty preference) contains evidential value, has potentially important implications for the interpretation of looking time experiments more generally. For one, it militates against drawing equivalence between novelty preferences and familiarity preferences; our results indicate that, at least within this dataset, novelty and familiarity preferences provide different evidential value, such that the less common response is more likely to be a false positive. Practically this means that, while there may be reasons for familiarity preferences in rule learning studies (e.g. due to differences in paradigm or infant age), such preferences should be treated with caution until replicated. In future work, it will be important to generalize this method, to see if the conclusion that only the more common behavioural response provides evidence also holds true for other phenomena. For example, most studies of infant word segmentation show a familiarity preference (Bergmann & Cristia, 2016); our finding would suggest that *novelty* preferences in that paradigm are likely to contain less evidential value.

## 8 CONCLUSION

Our combined meta‐analytic and experimental approaches suggest that infants best detect and generalize patterns from stimuli that are meaningfully relevant to their everyday experience with sounds, objects, and people. This provides support for recent trends in theories of development, and domain specificity in particular, that move away from broad cuts between domains, and towards more fine‐grained analyses of how the experiences of infants and children affect their learning. This trend is supported by both large‐scale observational work, such as on how children's linguistic and visual environments correlate with and perhaps support the development of their early vocabularies (Clerkin, Hart, Rehg, Yu, & Smith, 2017; Roy, Frank, DeCamp, Miller, & Roy, 2015), as well as by laboratory experiments that have scrutinized the influence of particular environmental features. For instance, infants segment words more easily from speech with more naturalistic distributional properties (Graf Estes & Lew‐Williams, 2015; Lew‐Williams, Pelucchi, & Saffran, 2011), learn sounds more easily from live interaction than from video (Kuhl, Tsao, & Liu, 2003), and recognize words more easily when spoken by their own mother rather than an unfamiliar voice (Parise & Csibra, 2012).

Like that work, our conclusions are based on both observational analyses and causal experimental tests, but in this case the observational analyses are themselves conducted over experimental results. We would argue that this combination of approaches provides important benefits. Meta‐analyses, like big data approaches, allow conclusions to be evaluated at scales that cannot be reached in typical lab experiments (Bergmann & Cristia, 2016; Cristia, 2018; Tsuji, Bergmann, & Cristia, 2014), while lab experiments complement the meta‐analyses by permitting direct causal tests of their conclusions. Converging evidence of this type should increasingly be part of the researchers’ arsenal given concerns about replicability and statistical power in psychology, and infant research in particular.

## ENDNOTES

1 A distinct but related literature, which we do not focus on here, has investigated how infants learn other types of rules (e.g. phonological and morphosyntactic; Gomez & Lakusta, 2004; Seidl & Buckley, 2005).

2 In the reverse regression, residualized meaningfulness significantly predicted infants’ behaviour above‐and‐beyond the effect of stimulus type (*p* = 0.03).

3 All gesture nomenclature is adopted from *American Sign Language Handshape Starter: A Beginner's Guide* (Tennant & Brown, 2002).

4 Although see Black and Bergmann (2017) and Conway and Christiansen (2005) for evidence that accuracy of transition probability learning is not uniform across stimulus types.
